# Synergistic Membrane Disruption of *E. coli* Tethered Lipid Bilayers by Antimicrobial Lipid Mixtures

**DOI:** 10.3390/biomimetics10110739

**Published:** 2025-11-04

**Authors:** Tun Naw Sut, Bo Kyeong Yoon, Joshua A. Jackman

**Affiliations:** 1 School of Chemical Engineering and Translational Nanobioscience Research Center, Sungkyunkwan University, Suwon 16419, Republic of Korea; suttunnaw@skku.edu; 2 School of Biomedical Engineering, Chonnam National University, Yeosu 59626, Republic of Korea; bkyoon@jnu.ac.kr

**Keywords:** antimicrobial lipid, monoglyceride, fatty acid, tethered bilayer lipid membrane, permeabilization, electrochemical impedance spectroscopy

## Abstract

Biomimetic lipid platforms provide versatile tools for mimicking various types of biological membranes and enable investigation of how industrially important amphiphiles (e.g., permeation enhancers and surfactants) interact with different membrane compositions. For example, antimicrobial lipids such as medium-chain fatty acids (FAs) and monoglycerides (MGs) are promising antibiotic alternatives that disrupt bacterial membranes and their distinct mechanisms of action are a topic of ongoing interest. The potency and targeting spectrum of individual antimicrobial lipids vary and mixing different lipids can improve functional activities. Biophysical studies indicate that optimally tuned mixtures exhibit greater disruption of synthetic lipid bilayers; however, their activity against more complex bacterial membrane compositions is largely unexplored. Herein, we applied electrochemical impedance spectroscopy (EIS) to investigate how two MG/FA pairs—composed of 10-carbon long monocaprin (MC) with capric acid (CA) and 12-carbon long glycerol monolaurate (GML) with lauric acid (LA)—disrupt tethered lipid bilayers composed of *Escherichia coli* bacterial lipids. While MC and CA individually inhibit *E. coli*, MC/CA mixtures at intermediate ratios displayed synergistic membrane-disruptive activity. Mechanistic studies showed that this synergistic activity depends on the MC/CA molar ratio rather than total lipid concentration. In contrast, GML/LA mixtures had weak membrane interactions across all tested ratios and lacked synergy, which is consistent with their low activity against *E. coli*. Together, the EIS results reveal that an effective disruption synergy against target membranes can arise from combining individually active antimicrobial lipids with distinct membrane-interaction profiles, laying the foundation to develop potent antimicrobial lipid formulations for tackling antibiotic-resistant bacteria.

## 1. Introduction

Biological membranes are quasi two-dimensional lipid assemblies that support various cellular functions such as compartmentalization, signaling, and protection, and provide inspiration for designing lipid-based biomaterial coatings and sensing platforms [1,2,3]. To mimic these features under controlled conditions, supported lipid bilayers (SLBs) and related membrane-mimicking architectures have been developed as experimental tools that recapitulate the self-assembly organization, molecular sealing, and interfacial behavior of biological membranes [4,5]. In conjunction with surface-sensitive measurement techniques, these biomimetic platforms enable quantitative investigation of how biomacromolecular interactions affect membrane structure, stability, and function, which is useful for testing membrane-active agents such as peptides and surfactants.

One promising class of membrane-active agents is antimicrobial lipids like fatty acids and monoglycerides, which are single-chain lipid amphiphiles that are promising solutions to overcome antibiotic resistance by inhibiting bacterial pathogens through membrane-disruptive mechanisms [6,7]. Saturated fatty acids (FAs) and monoglycerides (MGs) with medium-length hydrocarbon chains (6–12 carbons long) have especially high biological activity and are used for various applications, including healthcare, food science, and agriculture [8]. Many of them are Generally Recognized as Safe (GRAS) by the U.S. Food and Drug Administration and some have been reported to possess antibacterial activity at concentrations far below levels that affect mammalian cell viability (at least five-fold difference) [9]. Within this scope, it is increasingly recognized that different FAs and MGs have distinct targeting spectrums and potencies. For example, 10-carbon long capric acid (CA) and glycerol monocaprate (MC) are typically active against Gram-negative bacteria such as *E. coli* whereas 12-carbon long lauric acid (LA) and glycerol monolaurate (GML) mainly work against Gram-positive bacteria such as *S. aureus*. These inhibitory profiles have led to targeted use of specific antimicrobial lipids that work against certain bacterial types and have also spurred the development of antimicrobial lipid mixtures with expanded targeting spectrums [10,11].

To support these directions, biophysical studies have advanced molecular-level insights into how medium-chain FAs and MGs disrupt phospholipid membranes. Aided by surface-sensitive measurement techniques like quartz crystal microbalance-dissipation (QCM-D), localized surface plasmon resonance (LSPR), and fluorescence microscopy, real-time tracking of antimicrobial lipid interactions with membrane-mimicking supported lipid bilayer (SLB) platforms has identified that FAs and MGs cause distinct patterns of membrane disruption [12,13]. The differences in FA and MG interactions can be rationalized in part by their respective anionic and neutral headgroup properties that affect corresponding rates of membrane translocation and associated membrane strain profiles. These findings have been additionally supported by molecular dynamics (MD) simulations [14,15] and various biophysical studies have experimentally investigated the effects of factors such as antimicrobial lipid concentration, chain length, chain saturation, solution pH, membrane composition, and membrane geometry (e.g., SLB vs. intact vesicle adlayer) on antimicrobial lipid interactions [16,17]. A prevailing view is that medium-chain FAs and MGs mainly disrupt supported lipid membrane platforms above their specific critical micelle concentration (CMC) value, which fits with empirically observed potencies of different antimicrobial lipids and supports that self-assembled micelles play an important role in membrane disruption.

With growing knowledge about the physical chemistry of antimicrobial lipids, there is renewed opportunity to develop antimicrobial lipid mixtures based on combining the advantageous biophysical features of different FAs and MGs. It has been reported that co-mixing of FAs and MGs can markedly boost membrane-disruptive properties, as evidenced by 4-times greater disruption levels when an SLB platform was exposed to GML/LA mixed micelles as opposed to GML or LA micelles [18]. The most extensive membrane disruption occurred with an equimolar mixture of GML and LA (50/50 molar ratio), which was attributed to competing membrane morphological changes that were triggered by the distinct membrane-disruptive interactions of GML vs. LA molecules in the bilayer. In addition, it has been shown that 25/75 mol% GML/LA mixed micelles caused superior disruption of curved lipid vesicles compared to 50/50 mol% GML/LA mixed micelles, supporting that optimization of antimicrobial lipid mixtures depends on biophysical features of the membrane target [19]. Developing antimicrobial lipid mixtures that work well against Gram-negative bacterial lipid compositions would be particularly advantageous and recent demonstration of testing antimicrobial lipids with a tethered bilayer lipid membrane (tBLM) platform composed of an *E. coli* bacteria-derived lipid extract [20] provides analytical capabilities to achieve this goal.

In this study, we employed the electrochemical impedance spectroscopy (EIS) technique to investigate the membrane-disruptive effects of different antimicrobial lipid mixtures on *E. coli* bacteria-derived tBLM platforms and identified key insights into design parameters that affect activity levels. Two pairs of antimicrobial lipids were chosen that consisted of (1) 10-carbon MC monoglyceride and CA fatty acid that are known to be active against *E. coli* bacteria and (2) 12-carbon GML monoglyceride and LA fatty acid that are generally inactive against *E. coli* bacteria, and various molar ratios of the binary mixtures were tested systematically. The main readouts were time-resolved changes in membrane conductance (G_m_) and capacitance (C_m_), which are related to ionic permeability and structural integrity, respectively, and we sought to answer two biophysical-oriented questions. First, can mixing highly active MC and CA further improve membrane-disruptive activity against *E. coli* bacterial lipid-derived membranes, i.e., shifting from high to even higher performance? Second, can mixing weakly acting GML and LA enhance membrane-disruptive activity against *E. coli* bacterial lipid-derived membranes, i.e., shifting from low to high performance? 

## 2. Materials and Methods

Materials. The *E. coli* total lipid extract in chloroform (catalog no. 100500) was obtained from Avanti Polar Lipids, Inc. (Alabaster, AL, USA). The lipids were extracted from *E. coli* bacteria (ATCC 11303 strain) by dissolving them in a chloroform/methanol and precipitating against deionized water, and the extract corresponds to the concentrated phase in chloroform [21]. While the lipid extract was supplied in chloroform, dried films of the lipid extract were prepared using nitrogen air blowing and then re-dissolved in ethanol for experiments. Capric acid (CA), monocaprin (MC), and lauric acid (LA) were procured from Sigma Aldrich (St. Louis, MO, USA), and glycerol monolaurate (GML) was obtained from Abcam (Cambridge, UK). Deionized water with >18.2 MΩ·cm resistivity was prepared using a Milli-Q water purification system (MilliporeSigma, Burlington, MA, USA) and was used for diluting stock phosphate-buffered saline (PBS; 10×, pH 7.4) from Thermo Fisher Scientific (Waltham, MA, USA).

Sample Preparation. Stock solutions of CA, MC, LA, and GML (200 mM) were prepared in ethanol and diluted in PBS to obtain aqueous stocks. For concentration-based mixtures, aqueous stocks were prepared at twice the intended final concentration, diluted two-fold and then mixed in equal volumes. For ratio-based mixtures, individual aqueous stocks (8 mM total) were mixed to the desired ratio, heated at 70 °C for 30 min, cooled to room temperature, and diluted to the required test concentrations.

Electrochemical Impedance Spectroscopy (EIS). EIS measurements were conducted using the SDx tethaPOD instrument (SDx Tethered Membranes, Sydney, Australia) as previously described [22]. Glass slide-based sensor chips containing gold electrodes were pre-coated with a benzyl-disulfide ethylene glycol monolayer (10% tether, 90% spacer) [23]. tBLM platforms were prepared following established protocols based on rapid solvent-exchange [24]. Measurements were performed at a 25 mV alternating current (AC) with a swept frequency range of 0.1 to 2000 Hz to record EIS signals developed across the gold tethering electrodes and the gold reference electrodes embedded in the flow cells of the cartridge (two-terminal measurement system) [25]. The EIS data were fitted to an equivalent circuit model in the TethaQuick™ (version: v2.0.58) software package (see Figure 4A in Ref. [26]), which consists of a constant phase element (CPE) in series with a parallel resistor/CPE network by using a proprietary procedure based on the Levenberg–Marquardt algorithm [26] to determine changes in the conductance (G_m_) and capacitance (C_m_) properties of the tBLM platform. The procedure yielded optimized fit values and their relative fitting errors (Error %), which were satisfactorily low (<5%) in accordance with literature references [27,28] (see Table S1 in the Supplementary Information) and the absence of systematic deviations in the residual plots verified acceptable model fitting for all spectra.

## 3. Results and Discussion

### 3.1. Experimental Strategy

**Figure 1:**
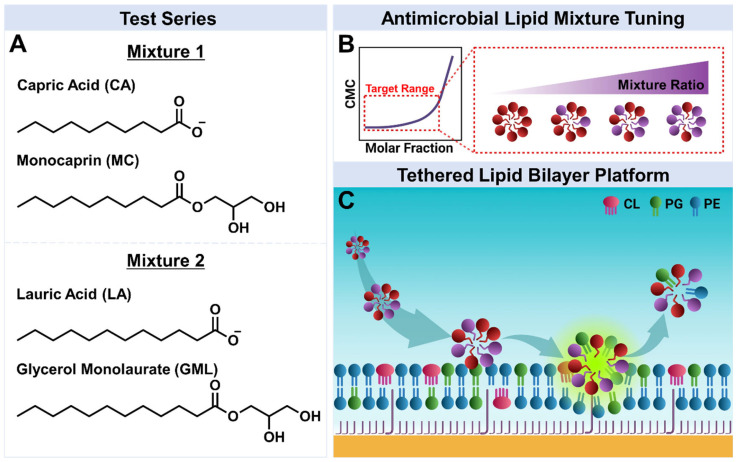
Experimental strategy. (**A**) Molecular structures of the tested antimicrobial lipids: mixtures of capric acid (CA) and monocaprin (MC), and mixtures of lauric acid (LA) and glycerol monolaurate (GML). (**B**) Optimizing antimicrobial lipid mixture ratios to improve potency based on CMC mapping of binary mixtures. (**C**) Tethered lipid bilayer membrane (tBLM) platform composed of cardiolipin (CL), phosphatidylglycerol (PG), phosphatidylethanolamine (PE), and other lipids.

Figure 1 presents the experimental strategy to characterize how different FA and MG mixtures disrupt *E. coli* bacterial membranes. Two sets of antimicrobial lipids composed of 10-carbon long MC monoglyceride and CA fatty acid (Mixture 1) and 12-carbon long GML monoglyceride and LA fatty acid (Mixture 2) were selected (Figure 1A). These mixtures were selected for two reasons. First, MC and CA are known to individually exhibit greater disruption of *E. coli* bacterial membranes compared to GML and LA, respectively [20]. Notably, 2000 µM MC was previously found to be more active than 2000 µM GML or LA in a direct comparison on *E. coli* lipid-derived tBLM platforms [20]. Second, GML and LA have demonstrated synergistic membrane disruption of more simplified, zwitterionic DOPC SLB platforms [18]. In the latter case, GML and LA were both independently active against DOPC lipid membranes and exhibited heightened activity when combined. Hence, exploring whether such synergistic activities can be achieved on more complex, biologically relevant membrane compositions relevant to bacterial pathogens is warranted.

When medium-chain, saturated FAs and MGs mix in binary pairs, they can exhibit ideal mixing that is mainly driven by hydrophobic interactions [29]. The CMC of the mixture depends on the respective CMC values of the FA and MG and on the molar ratio of the two components. For equivalent chain length pairs, MGs have lower CMCs by up to 5- to 10-fold due to nonionic headgroups that can readily self-assemble whereas corresponding FAs have higher CMCs due to typically anionic headgroups around physiological pH conditions [16]. Consequently, higher FA concentrations are needed to overcome the energy barrier of intermolecular repulsion between charged headgroups within the micellar structure [30]. Hence, mixtures with higher MG fractions tend to have lower CMCs mirroring the MG, which can translate into greater potency since surface-active micelles are the main membrane-disruptive species (Figure 1B). For this reason, we prepared mixed micelles in the range of 100/0 to 25/75 mol% MG/FA across different experimental formats to create a diverse range of mixtures while maximizing potency. We excluded pure FAs because they require appreciably higher concentrations that approach solubility limits and even moderate doping with MGs can markedly improve solubility and potency based on lower CMCs as discussed below.

The membrane-disruptive interactions of the MG/FA mixed micelles were characterized using *E. coli* bacterial lipid-derived tBLM platforms (Figure 1C). Extracts of *E. coli* bacterial lipids were composed of 9.8% cardiolipin (CL), 15.1% phosphatidylglycerol (PG), 57.5% phosphatidylethanolamine (PE) among various components, and were used to fabricate the tBLM platforms. All fabrication steps and subsequent interaction testing steps were characterized by EIS measurements, which temporally tracked changes in the conductance (G_m_) and capacitance (C_m_) properties of the tBLM adlayer. These two signals relate to ionic permeability and structural integrity (thickness and/or water content) of the tethered lipid bilayer, respectively. The sensitivity of the C_m_ signal to structural features of the tBLM is related to modeling the lipid bilayer as a parallel-plate capacitator [31]. Changes in membrane thickness affect the separation distance between the plates (i.e., greater distance yields less capacitance and vice versa) while changes in water content influence the relative permittivity of the dielectric medium between the two plates (i.e., greater water content yields greater capacitance) [32]. Notably, the *E. coli* membrane tBLM platform and its signal readouts have proven useful for characterizing the membrane-disruptive properties of an antimicrobial peptide series, which matched well with antibacterial activity levels against *E. coli* bacteria [33].

Using this measurement strategy, we fixed the bulk concentration of the main MG (MC or GML) and added varying concentrations of the corresponding FA (CA or LA) in one experimental series. Additionally, we fixed the total concentration of MG/FA at 2 × CMC and varied the MG/FA ratio systematically in another experimental series. These two series enabled us to dissect the relative contributions of total MG/FA concentration and MG/FA ratio to the membrane-disruptive activities of the respective mixed micelle system and elucidate key criteria that drive performance enhancement.

### 3.2. Antimicrobial Lipid Mixture Screening

**Figure 2:**
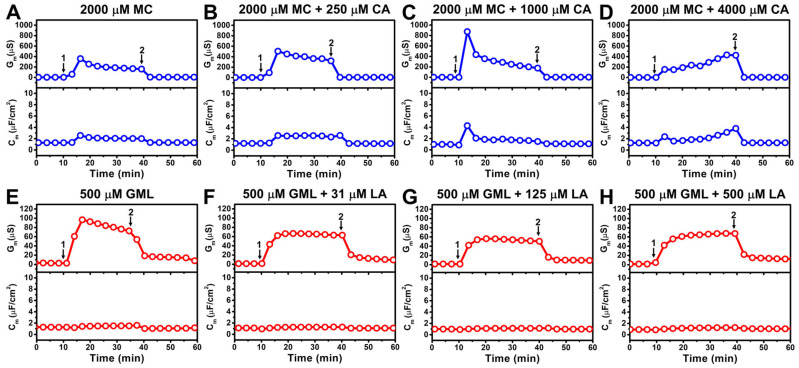
EIS screening of potential synergies between monoglyceride and fatty acid mixtures to disrupt *E. coli* lipid-derived tethered bilayers. Conductance (G_m_, upper panel) and capacitance (C_m_, lower panel) signals as a function of time for *E. coli* lipid-derived tBLM platforms due to interaction with monoglyceride and fatty acid mixtures. Two mixture series were investigated: C_10_ monoglyceride (MC) and fatty acid (CA) at (**A**) 2000 µM MC alone, (**B**) 2000 µM MC + 250 µM CA, (**C**) 2000 µM MC + 1000 µM CA, and (**D**) 2000 µM MC + 4000 µM CA; and C_12_ monoglyceride (GML) and fatty acid (LA) at (**E**) 500 µM GML alone, (**F**) 500 µM GML + 31 µM LA, (**G**) 500 µM GML + 125 µM LA, and (**H**) 500 µM GML + 500 µM LA. Baseline corresponds to fabricated *E. coli* lipid-derived tBLM platform and arrows 1 and 2 indicate mixture addition and buffer washing steps, respectively. Graphs are representative of *n* = 3 independent measurements.

An initial screen was performed to evaluate whether C_10_ and C_12_ MG and FA pairs exhibit synergistic membrane disruption. Since 10-carbon long MC is known to inhibit *E. coli* bacteria, we first investigated the effects of treating the *E. coli* tBLM platform with 2000 µM MC micelles (Figure 2A). Upon MC addition, there was rapid membrane disruption corresponding to ΔG_m_ and ΔC_m_ shifts around 360 µS and 2.6 µF/cm^2^, respectively.

The interaction kinetics indicate rapid destabilization within a few minutes and the ΔG_m_ and ΔC_m_ shift responses gradually stabilized at around 160 µS and 2.0 µF/cm^2^, respectively, during the interaction stage. A subsequent buffer washing step removed free MC from the bulk reservoir and led to major restoration of membrane integrity, as indicated by final ΔG_m_ and C_m_ shifts of 4 µS and 1.3 µF/cm^2^, respectively, that were closer to baseline values. These results support that the presence of MC micelles during the treatment stage (as defined by the bulk MC concentration at 2 × CMC) induced transient membrane disruption while effectively diluting the MC concentration in the bulk reservoir to below the CMC upon buffer washing markedly abrogated, but did not fully diminish, the membrane-disruptive effect.

As GML/LA mixtures were previously shown to trigger more extensive disruption of zwitterionic SLB platforms due to competing membrane morphological changes caused by MGs and FAs as described above, we proceeded to investigate whether adding mixtures of MC and CA—at a fixed MC concentration—could further enhance membrane disruption in the *E. coli* tBLM context. Since the CMC of CA by itself is around 3500 µM and elevated concentrations beyond that point approach practical solubility limits, we focused on initially adding a relatively small amount of 250 µM CA to 2000 µM MC to form MC/CA mixed micelles and then treated the *E. coli* tBLM with the mixture (Figure 2B). Although the molar fraction of CA was only around 11%, there was an appreciably greater membrane-disruptive effect compared to MC alone and the maximum ΔG_m_ shift was around 504 µS, which gradually decreased to around 320 µS. Hence, adding a small amount of CA led to a ~40% increase in the membrane ionic permeability during the peak disruption stage compared to MC alone while the ΔC_m_ shift responses were similar. Upon buffer washing, the final ΔG_m_ and ΔC_m_ shifts returned to around 6.3 µS and 1.2 µF/cm^2^, respectively, which demonstrates that 2000 µM MC + 250 µM CA addition also caused transient membrane disruption while a moderate degree of membrane permeabilization remained.

Interestingly, treatment with the 2000 µM MC + 1000 µM CA mixture—equivalent to 67 mol% MC and 33 mol% CA—caused markedly enhanced membrane disruption, with maximum ΔG_m_ and ΔC_m_ shifts peaking at around 875 µS and 4.3 µF/cm^2^, respectively (Figure 2C). Peak disruption occurred rapidly and the ΔG_m_ and ΔC_m_ shifts eventually stabilized at around 200 µS and 1.7 µF/cm^2^. Subsequent buffer washing caused the ΔG_m_ and ΔC_m_ shifts to decrease to around 3.2 µS and 1.1 µF/cm^2^, respectively, signaling that extensive membrane disruption occurred in the presence of MC/CA mixed micelles and the transient interaction was highly dynamic.

In marked contrast, treatment with the 2000 µM MC + 4000 µM CA mixture—reflecting a two-fold higher total MG/FA concentration and a reverse ratio of 33 mol% MC and 67 mol% CA—had distinct effects on the *E. coli* tBLM platform (Figure 2D). Initially, the ΔG_m_ shift response was around 154 µS and gradually increased to around 424 µS over the 30 min treatment period. The ΔC_m_ shift response also increased from around 2.4 µF/cm^2^ to 3.8 µF/cm^2^ over the same time interval and both ΔG_m_ and ΔC_m_ shifts returned to near-baseline values upon buffer washing. Despite a much higher total MC/CA concentration than the other cases, these results demonstrate that the 2000 µM MC + 4000 µM CA mixture had two major distinct effects as follows: (1) relatively small extent of initial membrane disruption; and (2) gradually increasing membrane disruption as opposed to rapid peak disruption. Together, these findings support that the molar ratio of MC and CA is a key determinant of enhancing the membrane-disruptive effect of MC and the most extensive disruption occurred for the 67/33 mol% MC/CA mixture.

In addition to MC as a base component, we proceeded to investigate the membrane-disruptive effects of 12-carbon long GML, which demonstrates high antibacterial potency against Gram-positive bacteria yet lacks significant inhibitory activity against Gram-negative bacteria such as *E. coli*. Since GML has a lower CMC than MC, we focused on testing the effects of treating an *E. coli* tBLM platform with 500 µM GML micelles. Compared to MC, GML treatment yielded a relatively smaller ΔG_m_ shift of around 100 µS that gradually decreased to around 60 µS over a 30 min time interval (Figure 2E). Subsequent addition of 31–500 µM LA to the 500 µM GML had negligible effect and the resulting GML/LA mixed micelles showed weaker membrane-disruptive activity, with ΔG_m_ shifts that stabilized at around 60 µS (Figure 2F–H). In all cases, the ΔC_m_ shifts were negligible, indicating that the overall structural integrity of the *E. coli* tBLM platforms remained largely intact upon GML/LA mixture treatment. This finding is striking because GML/LA mixtures, especially around a 50/50 molar ratio, have been reported to synergistically disrupt zwitterionic DOPC SLB platforms as described above. However, since GML and LA are largely inactive against *E. coli* bacterial lipid membranes, this result suggests that pairing MGs and FAs in well-defined mixtures can only enhance membrane disruption when the individual components themselves are active.

**Figure 3:**
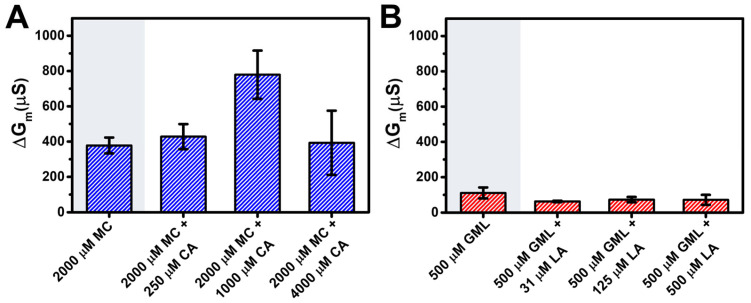
Comparison of maximum membrane conductance shifts for fixed monoglyceride concentrations. Summary of maximum conductance (ΔG_m_) shifts based on data in Figure 2. (**A**) MC/CA mixtures and (**B**) GML/LA mixtures. Shaded regions represent the shifts for MC and GML only controls. Mean and standard deviation are reported from *n* = 3 measurements.

Figure 3 summarizes the maximum ΔG_m_ shift responses that are indicative of changes in membrane ionic permeability. While 2000 µM MC treatment resulted in a maximum ΔG_m_ shift of 378 ± 45 µS, 2000 µM MC + 250 µM CA and 2000 µM MC + 1000 µM CA treatments yielded larger shifts of 428 ± 71 µS and 779 ± 137 µS, respectively (Figure 3A). This trend indicates that the 2000 µM MC + 1000 µM CA treatment triggered nearly 2-fold higher ΔG_m_ shifts than the other tested compositions, which included mixtures with either higher or lower total MC/CA concentrations.

However, notably, the 2000 µM MC + 4000 µM CA treatment yielded a smaller maximum ΔG_m_ shift of 393 ± 182 µS despite an appreciably higher total MC/CA concentration. In other words, CA addition enhances the membrane-disruptive activity of MC up to an intermediate CA concentration but its potentiating effect was diminished, and to some extent counteracted, at higher CA concentrations. This trend suggests a ratio-dependent effect although other possibilities exist like solubility limits as well. Conversely, for GML/LA mixtures, the maximum ΔG_m_ shift of 500 µM GML was 111 ± 31 µS and decreased to around 60–70 µS for 500 µM GML plus 31–500 µM LA (Figure 3B). This trend indicates that LA addition has a modest diminishing effect on the membrane-disruptive activity of GML across all tested LA concentrations.

### 3.3. Mechanistic Comparison of Antimicrobial Lipid Mixtures

**Figure 4:**
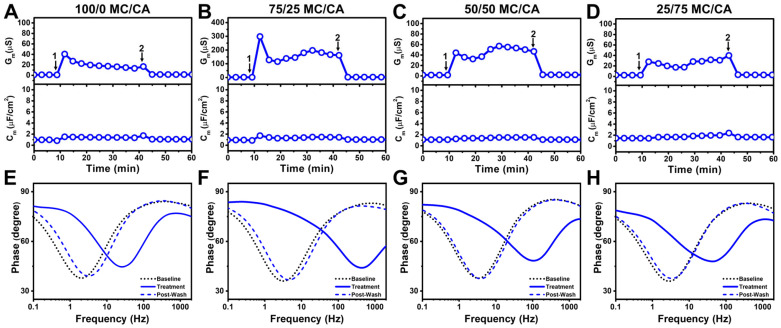
EIS characterization of MC/CA mixtures to inhibit *E. coli* lipid-derived tethered bilayers. Conductance (G_m_, upper panel) and capacitance (C_m_, lower panel) signals as a function of time for *E. coli* lipid-derived tBLM platforms due to interaction with MC/CA mixtures at (**A**) 100/0 mol%, (**B**) 75/25 mol%, (**C**) 50/50 mol%, and (**D**) 25/75 mol% ratios. All mixtures were tested at 2 × CMC of the binary mixture. Baseline corresponds to fabricated *E. coli* lipid-derived tBLM platform and arrows 1 and 2 indicate mixture addition and buffer washing steps, respectively. (**E**–**H**) Corresponding Bode phase plots for each case showing ‘Baseline’ (stable signal before treatment, arrow 1), ‘Treatment’ (spectrum immediately before washing, arrow 2), and ‘Post-Wash’ (spectrum after washing), were obtained by sweeping the frequency at 3 min intervals. Graphs are representative of *n* = 3 independent measurements.

To resolve the effects of molar ratio on membrane-disruptive behavior in the micelle regime, we next tested mixtures that were prepared at 2 × CMC whereby the respective CMC of each mixture was defined based on ideal mixing of the input components. This approach enabled us to ensure micelle formation while systematically varying the MG/FA ratio in 25 mol% increments. We first tested the effects of 100 mol% MC treatment (equivalent to 1200 µM concentration) and observed an initial ΔG_m_ shift of 40 µS that reached around 18 µS and returned to near-baseline values upon buffer washing (Figure 4A). In marked contrast, 75/25 mol% MC/CA treatment (equivalent to 1520 µM concentration) led to a markedly larger ΔG_m_ shift spike around 300 µS that dipped and fluctuated around 150–200 µS (Figure 4B). On the other hand, the addition of 50/50 mol% MC/CA (equivalent to 2000 µM concentration) and 25/75 mol% MC/CA (equivalent to 3200 µM concentration) led to smaller ΔG_m_ shifts around 40–60 µS (Figure 4C,D). These results reinforce that the MC/CA molar ratio, rather than the total MC/CA concentration, is the main driver of enhancing membrane disruption provided the MC/CA mixture is in the micellar concentration range.

Notably, in all cases, there were minimal ΔC_m_ shifts indicating negligible membrane thinning and membrane permeabilization was the main interaction event in this MC/CA concentration regime. The corresponding Bode phase plot shifts provide further insight into the extent of membrane disruption and support that the 75/25 mol% MC/CA mixture caused the greatest changes in membrane integrity based on the frequency-at-minimum-phase shift while all tested compositions caused at least moderate damage during the treatment step and the damage was mostly reversible upon buffer washing (Figure 4E–H). Thus, among all tested ratios at 2 × CMC, the 75/25 MC/CA mixture uniquely caused enhanced membrane permeabilization during treatment, supporting that an intermediate ratio of MC and CA components maximizes membrane-disruptive activity.

**Figure 5:**
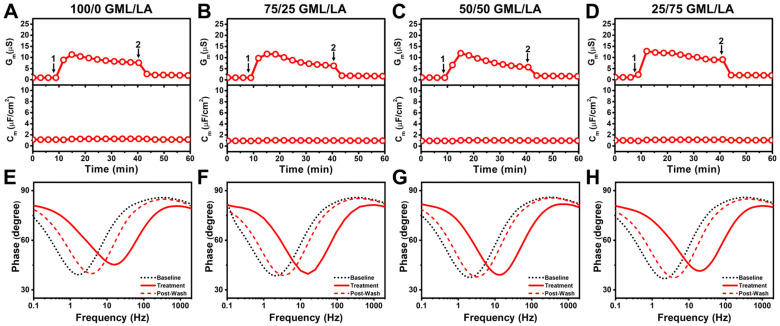
EIS characterization of GML/LA mixtures to inhibit *E. coli* lipid-derived tethered bilayers. Conductance (G_m_, upper panel) and capacitance (C_m_, lower panel) signals as a function of time for *E. coli* lipid-derived tBLM platforms due to interaction with GML/LA mixtures at (**A**) 100/0 mol%, (**B**) 75/25 mol%, (**C**) 50/50 mol%, and (**D**) 25/75 mol% ratios. All mixtures were tested at 2 × CMC of the binary mixture. Baseline corresponds to fabricated *E. coli* lipid-derived tBLM platform and arrows 1 and 2 indicate mixture addition and buffer washing steps, respectively. (**E**–**H**) Corresponding Bode phase plots for each case showing ‘Baseline’ (stable signal before treatment, arrow 1), ‘Treatment’ (spectrum immediately before washing, arrow 2), and ‘Post-Wash’ (spectrum after washing), were obtained by sweeping the frequency at 3 min intervals. Graphs are representative of *n* = 3 independent measurements.

In addition, we tested the effects of 100 mol% GML treatment (equivalent to 160 µM concentration) and observed an initial ΔG_m_ shift of 15 µS that gradually declined to around 10 µS and returned to near-baseline values upon buffer washing (Figure 5A). There were also negligible ΔG_m_ shifts throughout the interaction process. Nearly identical ΔG_m_ and ΔC_m_ shifts were observed in the cases of 75/25, 50/50, 25/75 mol% GML/LA mixtures that were equivalent to 200, 320, and 520 µM concentrations, respectively (Figure 5B–D). The corresponding Bode phase plots showed similar levels of membrane disruption across the tested GML/LA mixtures and frequency-at-minimum-phase shifts were markedly smaller than in the 75/25 and 50/50 mol% MC/CA mixture cases, while the shifts largely recovered upon buffer washing (Figure 5E–H).

As controls, we also tested the same MC/CA and GML/LA mixtures at 0.5 × CMC and observed negligible membrane-disruptive effects in all cases (Supplementary Figures S1 and S2). This finding supports that mixed micelles are the main species of MC/CA and GML/LA mixtures that disrupt *E. coli* membranes.

**Figure 6:**
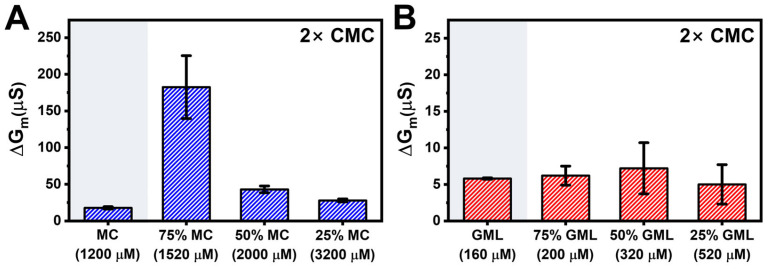
Comparison of maximum membrane conductance shifts for varying monoglyceride/fatty acid ratios at 2× CMC. Summary of maximum conductance (ΔG_m_) shifts based on data in Figure 4 and Figure 5. (**A**) MC/CA mixtures and (**B**) GML/LA mixtures. Shaded regions represent the shifts for MC and GML only controls. Listed values below each column indicate total concentration of antimicrobial lipids. Mean and standard deviation are reported from *n* = 3 measurements.

Figure 6 summarizes the maximum ΔG_m_ shift responses for the different MC/CA and GML/LA mixture cases at 2 × CMC. The 75/25 mol% MC/CA micelles yielded the largest ΔG_m_ shifts around 183 ± 43 µS whereas the other mixed micelle compositions caused ΔG_m_ shifts of less than 50 µS (Figure 6A). Likewise, all GML/LA micelle compositions induced ΔG_m_ shifts of less than 15 µS (Figure 6B). Together, these findings support that MC/CA mixtures can display synergistic membrane disruption of *E. coli* lipid-derived tBLM platforms whereas GML/LA mixtures have weak activity overall. These findings also point to the importance of MC/CA molar ratio within the micelle-forming concentration regime rather than the absolute total MC/CA concentration in modulating the extent of membrane-disruptive activity.

As noted above, the G_m_ shifts returned to near-baseline values after buffer washing, which supports that membrane disruption is largely reversible. This finding is consistent with initial partitioning of antimicrobial lipid molecules into the tBLM platform that leads to transient packing defects and hence greater membrane permeability. Around CMC, antimicrobial lipid–membrane interactions result in coexistence of lamellar-phase bilayers and mixed micelles whereby partial membrane solubilization can occur [34]. However, the effects are still relatively modest [35] and largely reversible upon buffer washing since the medium-chain antimicrobial lipids, which have short residence times in membranes, can revert from the membrane-embedded state to the bulk phase. This behavior is further supported by the known function of CA as a permeation enhancer that transiently perturbs lipid bilayers without causing irreversible lysis [36,37]. Consequently, the observed synergy of MC/CA pairs represents a delicate balance between partial membrane solubilization and membrane recovery upon antimicrobial lipid removal.

To rationalize the distinct effects of MC/CA vs. GML/LA pairs, we note that medium-chain FAs and MGs have shorter hydrocarbon chains compared to the phospholipids (mainly C_16_ to C_18_ chains; see Ref. [21]) that are found in the *E. coli* total lipid extract. Upon insertion into *E. coli* lipid bilayers, the antimicrobial lipids with shorter chains locally reduce the effective membrane thickness due to hydrophobic mismatch [38]. This hydrophobic mismatch and resulting effects on membrane structure (e.g., thinning and weakening) contribute to membrane destabilization and defect formation. Within this context, CA and MC have shorter chains than LA and GML, leading to greater hydrophobic mismatch and hence more extensive membrane disruption.

In agreement with this conceptual framework, all tested FAs and MGs have previously been reported to disrupt synthetic DOPC lipid bilayers [12,39]. Among them, MC had particularly high permeabilizing activity, which aligns with its shorter chain length [39]. Separately, it has been reported that GML/LA mixtures exhibit greater membrane-disruptive activity than GML or LA alone due to competing membrane morphological changes [18]. More specifically, GML induces membrane budding whereas LA triggers membrane tubulation and the interplay of these remodeling processes can lead to pearling instability and heightened membrane disruption.

Beyond model membranes, antibacterial experiments against Gram-positive bacteria such as *Staphylococcus aureus* have demonstrated that LA and CA are both active while LA is typically regarded as more potent due to a lower CMC [40]. Similarly, GML and MC are also both active against *S. aureus* and are more potent than their corresponding FAs due to lower CMCs [40]. Certain GML/LA mixtures have also been reported to inhibit Gram-positive *Streptococcus pyogenes* bacteria more than GML or LA alone [41].

On the other hand, only MC and CA display significant antibacterial activity against Gram-negative *E. coli* bacteria [42,43,44,45] whereas GML and LA are considered inactive [42,43,46,47,48]. It has also been verified that CA induces greater permeabilization of *E. coli* membranes than LA in vitro [49] and that MC and CA individually disrupt *E. coli* membranes more than GML and LA, respectively [20]. These findings support that only MC and CA cause sufficiently high levels of membrane disruption to damage *E. coli* membranes, which fits with their smaller packing parameters and corresponding greater curvature-inducing effects than GML and LA. In other words, GML and LA can destabilize relatively simplified membranes with high potency but are less effective against more complex ones such as *E. coli* membranes with rigidified components. Conversely, MC and CA have greater destabilizing effects, which are sufficiently strong to damage *E. coli* membranes.

**Figure 7:**
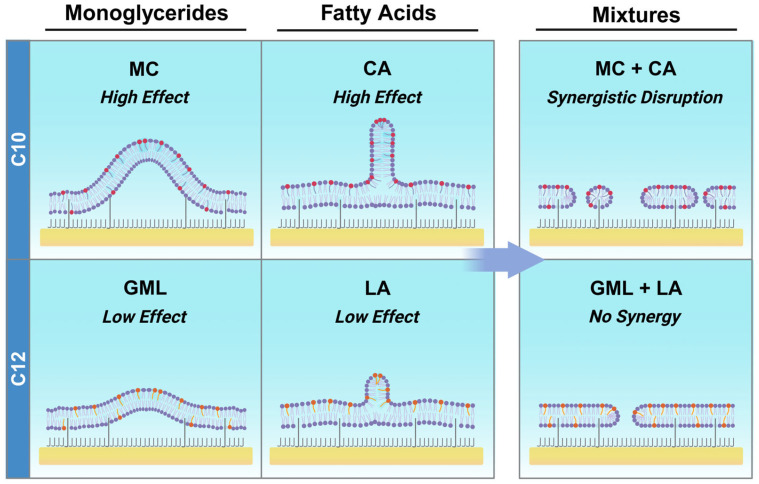
Schematic comparison of MC/CA and GML/LA mixture effects on tethered *E. coli* lipid bilayers. Individually, MC and CA have distinct, large interaction effects on *E. coli* membranes that induce synergistic membrane disruption due to competing membrane morphological changes. GML and LA also exhibit distinct interaction effects but the corresponding magnitudes are smaller so synergistic membrane disruption is not observed. The mixture schematics represent the treatment step when the tethered *E. coli* lipid bilayers are exposed to antimicrobial lipid mixtures and illustrate the relative degree of membrane permeabilization for each mixture. The depicted packing defects indicate reversible membrane permeabilization rather than stable pore formation.

The present experiments expand on these past observations by demonstrating that MC/CA mixtures can synergistically disrupt *E. coli* membranes (Figure 7). This behavior originates from the ability of MC and CA to individually inhibit *E. coli* bacteria, i.e., inducing a significantly strong membrane-damaging effect, and their distinct membrane-interaction behaviors whereby MC and CA are known to induce membrane budding and tubulation, respectively. In addition to differences in their molecular lengths, these distinct interaction behaviors are partially related to the distinct charge properties of MC and CA, whereby nonionic MC can readily translocate across the two bilayer leaflets and anionic CA is mainly found in the outer leaflet and has a higher energy barrier to flip into the inner leaflet [14]. Accordingly, MC and CA insertion cause different membrane strain profiles, which lead to their distinct membrane-interaction behaviors and hence competing membrane morphological changes.

In this regard, MC is particularly active, which fits with our observation that MC/CA mixtures with relatively high MC fractions (67–75 mol%) had the greatest permeabilizing effect. Conversely, GML and LA are non-inhibitory against *E. coli*, which is consistent with weaker membrane interactions observed in this study. Hence, even though GML and LA have unique membrane-interaction behaviors largely akin to MC and CA, the extents to which GML and LA disrupt *E. coli* membranes are below threshold levels needed for GML/LA mixtures to trigger synergistic membrane disruption.

Lastly, it is important to discuss the observation that the MC/CA mixtures exhibit reversible membrane disruption whereby large electrochemical changes in membrane properties are observed during the treatment step but mainly recover to near-baseline values upon buffer washing. From an application perspective, the treatment step itself, i.e., when the bacterial membrane composition is exposed to antimicrobial lipids, is most relevant to biological usage, which motivates further exploration of antibacterial activity levels in future studies. Indeed, while certain MC/CA mixtures showed optimal activity to disrupt *E. coli* lipid-derived tBLMs, additional research is needed to investigate their effects against different types of biological membranes, including potential selectivity and cytotoxicity assessment. It should be noted that GML/LA mixtures previously exhibited synergistic activity against synthetic DOPC lipid bilayers bearing similarity to the phosphatidylcholine (PC) lipids found in mammalian plasma membranes but lacked synergy against *E. coli* lipid-derived tBLM platforms observed herein. This distinction supports that the MC/CA mixtures might be similarly selective towards certain types of membrane compositions.

## 4. Conclusions

In summary, our findings highlight how synergistic membrane disruption of *E. coli* membranes benefits from rationally selecting antimicrobial lipids that are individually active and possess distinct membrane interaction behaviors, leading to competing membrane morphological changes that culminate in extensive disruption. It should also be emphasized that synergistic membrane disruption requires selecting antimicrobial lipids that have distinct membrane-interaction behaviors. For example, it has been previously reported that GML/MC mixtures comprising two MGs with similar molecular properties and membrane-interaction behaviors lacked synergy against DOPC lipid bilayers [50]. As such, balancing the interplay of antimicrobial lipid activity, potency, and mechanism is critical to developing high-performing mixtures to target biologically relevant membrane compositions.

Looking forward, such strategies can be combined with potency enhancement methods like doping antimicrobial lipid mixtures with other lipophilic agents that have low CMCs to further boost performance and open the door to various applications in the antimicrobial mitigation and therapeutic spaces. In terms of future studies, our findings motivate further exploration of the antibacterial activity of antimicrobial lipid mixtures against different types of Gram-negative and Gram-positive bacteria as well as the potential to encapsulate other classes of antimicrobial drugs (e.g., antibiotics) within the micellar assemblies to aid permeabilization-mediated delivery. Such possibilities fit within the scope of certain antimicrobial lipids such as CA functioning as permeation enhancers and could further lead to diverse uses of antimicrobial lipid technology for not only antibacterial mitigation but also for immunomodulation and drug delivery. In addition to tBLM platforms, expanding research efforts on antimicrobial lipid technologies to other types of model membrane platforms could open the door to a wider range of complementary surface-sensitive measurement techniques such as atomic force microscopy and fluorescence microscopy as well.

## Data Availability

The raw data required to reproduce these findings are available from the corresponding author on reasonable request.

## Supplementary Materials

The following supporting information can be downloaded at: https://www.mdpi.com/article/10.3390/biomimetics10110739/s1, Supplementary Figure S1: EIS characterization of MC/CA monomer mixtures to inhibit *E. coli* lipid-derived tethered bilayers; Supplementary Figure S2: EIS characterization of GML/LA monomer mixtures to inhibit *E. coli* lipid-derived tethered bilayers; Supplementary Table S1. Relative fitting errors (Error %) for membrane conductance (G_m_) and capacitance (C_m_) obtained from EIS analysis during each experimental stage.
