# Supplementary material for: Synergistic Membrane Disruption of E. coli Tethered Lipid Bilayers by Antimicrobial Lipid Mixtures
Source: Biomimetics (Basel). 2025 Nov 4;10(11):739. doi: 10.3390/biomimetics10110739 (PMC12650487; doi:10.3390/biomimetics10110739)
Supplement: Supplementary file 1 [file biomimetics-10-00739-s001.zip › biomimetics-3912093-supplementary.pdf]

biomimetics

MDPI

Supplementary Information

Synergistic Membrane Disruption of *E. coli* Tethered Lipid Bilayers by Antimicrobial Lipid Mixtures

Tun Naw Sut [1,†], Bo Kyeong Yoon [2,†] and Joshua A. Jackman [1,*]

[1] School of Chemical Engineering and Translational Nanobioscience Research Center, Sungkyunkwan University, Suwon 16419, Republic of Korea; suttunnaw@skku.edu (T.N.S.)
[2] School of Biomedical Engineering, Chonnam National University, Yeosu 59626, Republic of Korea; bkyoon@jnu.ac.kr (B.K.Y.)
† These authors contributed equally to this work.
* Correspondence: jjackman@skku.edu (J.A.J.)

Supplementary Figures

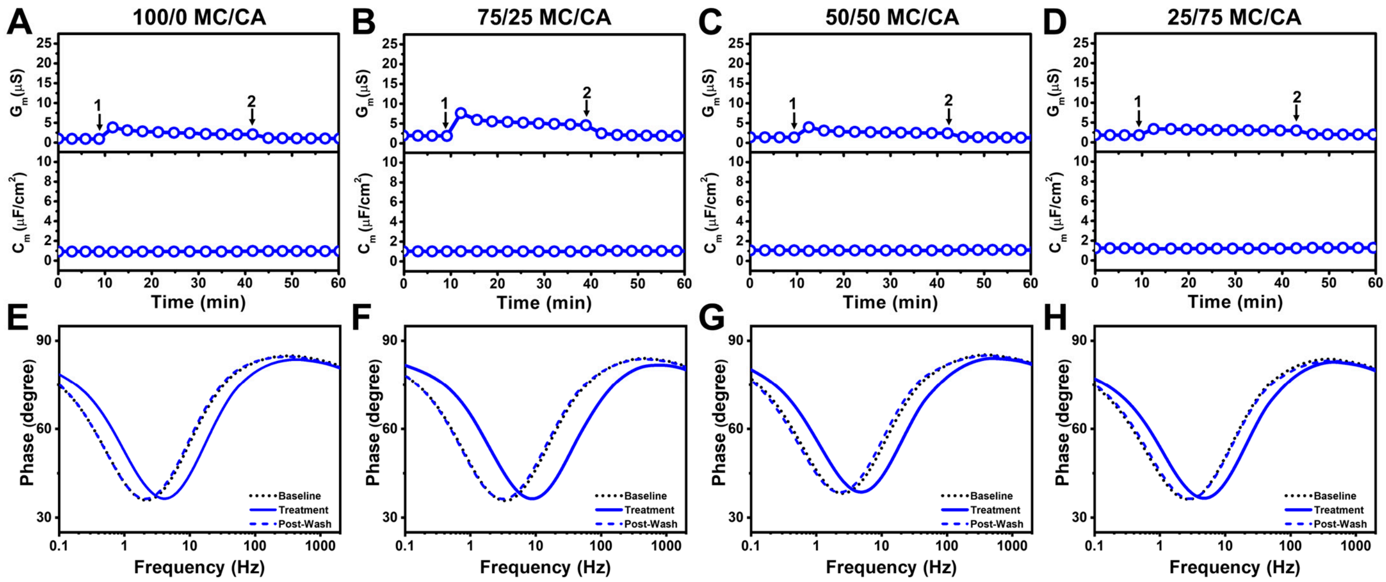

Supplementary Figure S1. EIS characterization of MC/CA monomer mixtures to inhibit *E. coli* lipid-derived tethered bilayers. Conductance (G_m, upper panel) and capacitance (C_m, lower panel) signals as a function of time for *E. coli* lipid-derived tBLM platforms due to interaction with MC/CA mixtures at **(A)** 100/0 mol%, **(B)** 75/25 mol%, **(C)** 50/50 mol%, and **(D)** 25/75 mol% ratios. All mixtures were tested at 0.5×CMC of the binary mixture. Baseline corresponds to fabricated *E. coli* lipid-derived tBLM platform and arrows 1 and 2 indicate mixture addition and buffer washing steps, respectively. **(E-H)** Corresponding Bode phase plots for each case showing 'Baseline' (stable signal before treatment, arrow 1), 'Treatment' (spectrum immediately before washing, arrow 2), and 'Post-Wash' (spectrum after washing), were obtained by sweeping the frequency at 3 min intervals. Graphs are representative of *n*=3 independent measurements.

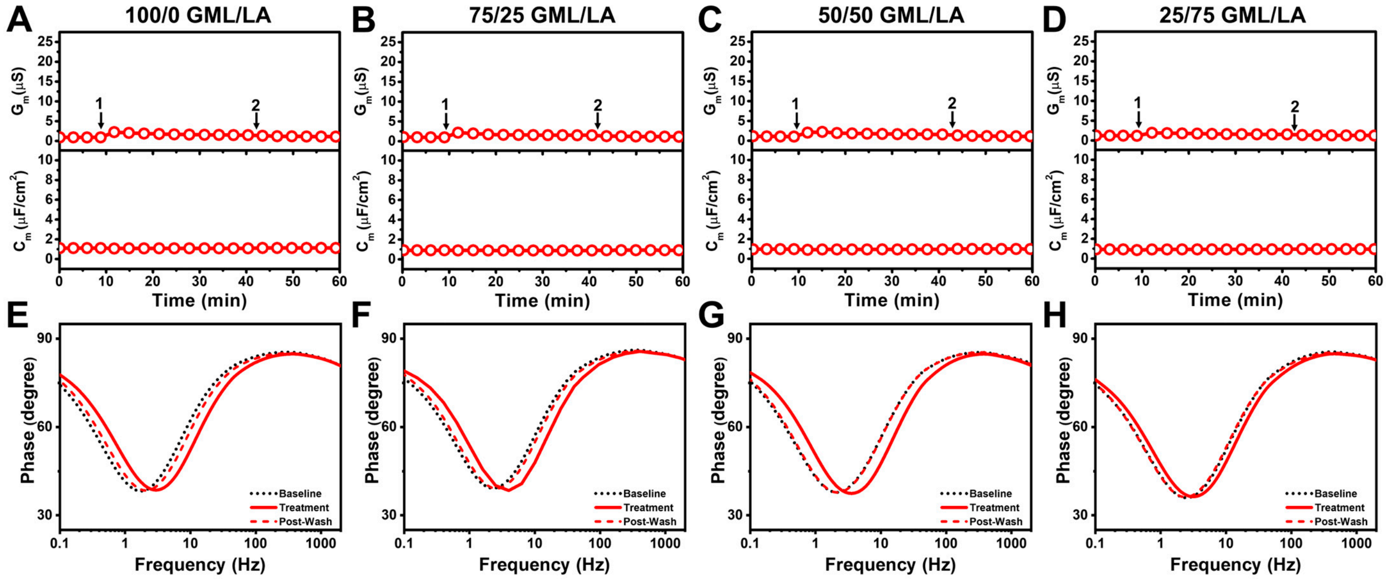

Supplementary Figure S2. EIS characterization of GML/LA monomer mixtures to inhibit *E. coli* lipid-derived tethered bilayers. Conductance (G_m, upper panel) and capacitance (C_m, lower panel) signals as a function of time for *E. coli* lipid-derived tBLM platforms due to interaction with GML/LA mixtures at **(A)** 100/0 mol%, **(B)** 75/25 mol%, **(C)** 50/50 mol%, and **(D)** 25/75 mol% ratios. All mixtures were tested at 0.5×CMC of the binary mixture. Baseline corresponds to fabricated *E. coli* lipid-derived tBLM platform and arrows 1 and 2 indicate mixture addition and buffer washing steps, respectively. **(E-H)** Corresponding Bode phase plots for each case showing 'Baseline' (stable signal before treatment, arrow 1), 'Treatment' (spectrum immediately before washing, arrow 2), and 'Post-Wash' (spectrum after washing), were obtained by sweeping the frequency at 3 min intervals. Graphs are representative of *n*=3 independent measurements.

Supplementary Table

MG/FA Mixture	Experimental Stage	Fitting Error% for G_m	Fitting Error% for C_m
100/0 MC/CA 2×CMC	Baseline	1.52	1.12
	Treatment	1.93	1.32
	Post-Wash	1.06	0.71
75/25 MC/CA 2×CMC	Baseline	2.27	1.55
	Treatment	2.68	4.08
	Post-Wash	2.08	1.36
50/50 MC/CA 2×CMC	Baseline	1.77	1.21
	Treatment	2.69	1.95
	Post-Wash	1.68	1.11
25/75 MC/CA 2×CMC	Baseline	2.51	1.86
	Treatment	2.87	2.02
	Post-Wash	1.59	1.1
100/0 GML/LA 2×CMC	Baseline	1.25	0.96
	Treatment	1.94	1.34
	Post-Wash	1.06	0.73
75/25 GML/LA 2×CMC	Baseline	1.27	0.92
	Treatment	1.89	1.35
	Post-Wash	1.37	0.95
50/50 GML/LA 2×CMC	Baseline	1.24	0.86
	Treatment	1.71	1.18
	Post-Wash	1.15	0.76
25/75 GML/LA 2×CMC	Baseline	1.01	0.95
	Treatment	1.43	0.98
	Post-Wash	1.08	0.75
100/0 MC/CA 0.5×CMC	Baseline	1.49	1.08
	Treatment	1.7	1.13
	Post-Wash	1.66	1.24
75/25 MC/CA 0.5×CMC	Baseline	1.93	1.38
	Treatment	2.05	1.4
	Post-Wash	2.13	1.53
50/50 MC/CA 0.5×CMC	Baseline	1.92	1.39
	Treatment	1.98	1.37
	Post-Wash	2.14	1.61
25/75 MC/CA 0.5×CMC	Baseline	2.05	1.59
	Treatment	2.11	1.46
	Post-Wash	2.52	1.93
100/0 GML/LA 0.5×CMC	Baseline	1.34	1.03
	Treatment	1.41	0.99
	Post-Wash	1.44	1.08

75/25 GML/LA 0.5×CMC	Baseline	1.09	0.78
	Treatment	1.09	0.71
	Post-Wash	1.25	0.82
50/50 GML/LA 0.5×CMC	Baseline	1.01	0.82
	Treatment	0.63	0.48
	Post-Wash	0.97	0.8
25/75 GML/LA 0.5×CMC	Baseline	1.3	0.97
	Treatment	1.28	0.88
	Post-Wash	1.41	1.01

Supplementary Table S1. Relative fitting errors (Error %) for membrane conductance (G_m) and capacitance (C_m) obtained from EIS analysis during each experimental stage. The stages correspond to Baseline (before treatment), Treatment (during MC/CA or GML/LA mixture exposure), and Post-Wash (after buffer washing). The data correspond to representative graphs plotted in the main text.
